# Staggered Chromosomal Hybrid Zones in the House Mouse: Relevance to Reticulate Evolution and Speciation

**DOI:** 10.3390/genes1020193

**Published:** 2010-07-19

**Authors:** İslam Gündüz, Christianne L. Pollock, Mabel D. Giménez, Daniel W. Förster, Thomas A. White, Maria A. Sans-Fuentes, Heidi C. Hauffe, Jacint Ventura, María José López-Fuster, Jeremy B. Searle

**Affiliations:** 1 Department of Biology, University of York, PO Box 373, York YO10 5YW, UK; E-Mails: gunduzi@omu.edu.tr (İ.G.); christianne.pollock@pms.ac.uk (C.L.P.); mdg500@york.ac.uk (M.D.G.); dwgfoerster@googlemail.com (D.W.F.); heidi.hauffe@iasma.it (H.C.H.); 2 Department of Biology, Faculty of Arts and Sciences, University of Ondokuz Mayis, Samsun, Turkey; 3 School of Biological and Biomedical Sciences, Durham University, Durham DH1 3LE, UK; E-Mail: tawhite201@hotmail.com; 4 Department of Ecology and Evolutionary Biology, University of Arizona, Tucson, AZ 85721, USA; E-Mail: sans@email.arizona.edu; 5 Departament de Biologia Animal, Facultat de Biologia, Universitat de Barcelona, Avda. Diagonal 645, 08028 Barcelona, Spain; E-Mail: marialopez@ub.edu (M.J.L.-F.); 6 Fondazione Edmund Mach, Centre for Research and Innovation, Environment and Natural Resources Area, Via E. Mach 1, 38010 S. Michele all’Adige (TN), Italy; 7 Departament de Biologia Animal, de Biologia Vegetal i d’Ecologia, Facultat de Biociènces, Universitat Autònoma de Barcelona, 08193 Bellaterra, Spain; E-Mail: jacint.ventura.queija@uab.es

**Keywords:** clines, *Mus musculus domesticus*, raciation, Robertsonian fusions, speciation

## Abstract

In the house mouse there are numerous chromosomal races distinguished by different combinations of metacentric chromosomes. These may come into contact with each other and with the ancestral all-acrocentric race, and form hybrid zones. The chromosomal clines that make up these hybrid zones may be coincident or separated from each other (staggered). Such staggered hybrid zones are interesting because they may include populations of individuals homozygous for a mix of features of the hybridising races. We review the characteristics of four staggered hybrid zones in the house mouse and discuss whether they are examples of primary or secondary contact and whether they represent reticulate evolution or not. However, the most important aspect of staggered hybrid zones is that the homozygous populations within the zones have the potential to expand their distributions and become new races (a process termed ‘zonal raciation’). In this way they can add to the total ‘stock’ of chromosomal races in the species concerned. Speciation is an infrequent phenomenon that may involve an unusual set of circumstances. Each one of the products of zonal raciation has the potential to become a new species and by having more races increases the chance of a speciation event.

## 1. Introduction

**Figure 1:**
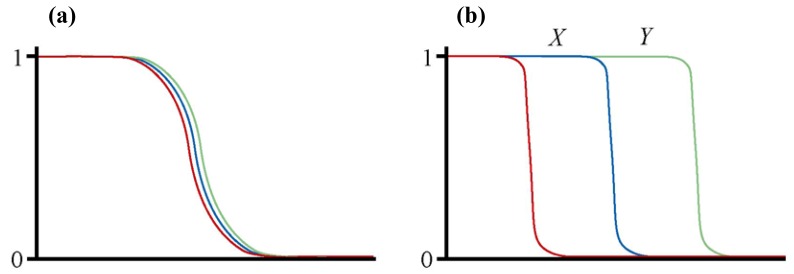
Diagrams illustrating the difference between a hybrid zone with coincident **(a)** and staggered **(b)** clines. In each graph the x axis represents geographic distance along a transect across the hybrid zone and the y axis represents frequency. Each coloured line represents the cline for a different genetic character. See text for further details.

When races are in contact and hybridising, the geographic area where hybrids are found is known as a ‘hybrid zone’ [1]. Such races are defined as genetically distinct forms within species, and there may be multiple genetic characters that differ between the races. Therefore, within a hybrid zone multiple character clines would be expected in the progression from one race to the other [2]. Those clines may be coincident within the hybrid zone, *i.e.*, centred at the same place in transects across the hybrid zone, or staggered, *i.e.*, centred at different places (Figure 1) [3].

Staggered hybrid zones have the interesting property that there may be homozygous types present within the hybrid zone that are different from either of the hybridising races. In the example given in Figure 1b, the region *X* of the hybrid zone is characterised by individuals which are homozygous both for one character that defines the hybridising race at the right end of the transect and for two characters that define the hybridising race at the left end of the transect. Therefore, a homozygous type with characteristics of both hybridising races is present within the staggered hybrid zone. Through later range expansion this homozygous type could have a sufficiently large distribution to be considered a new race (the same could happen to the second homozygous type that is present in region *Y* of the hybrid zone: Figure 1). This process whereby new races originate in hybrid zones has been termed ‘zonal raciation’ [4]. Clearly, a new race generated in this way may go on to become a new species.

So, how does this relate to reticulate evolution? If the two hybridising races represented in Figure 1b have come into secondary contact, then the homozygous types that occur at high frequency in regions *X* and *Y* would be some sort of mixture of two previously independent genomes, and therefore would be products of reticulate evolution. However, the staggering of clines could also represent a primary contact. In this case, the homozygous types in regions *X* and *Y* could be viewed as earlier stages in the formation of one of the hybridising races, so in that sense are not derived from two previously independent genomes and are therefore not the products of reticulate evolution.

**Figure 2:**
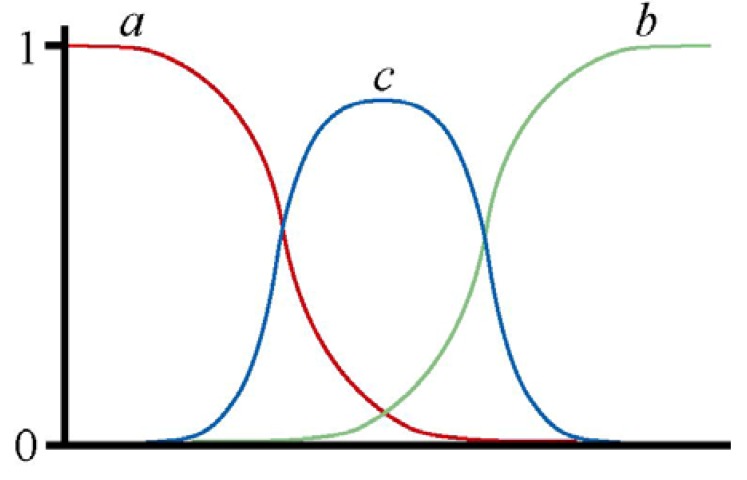
Diagram illustrating staggered clines within a hybrid zone relating to alleles at one locus. The x axis represents geographic distance along a transect across the hybrid zone and the y axis represents frequency. One hybridising race is characterised by allele *a*, and the other by allele *b*. A new allele (*c*) arises within the hybrid zone and its high frequency causes staggering of the clines for the race-specific alleles.

There is a third way to have staggered clines: that is within a single locus, if a new allele arises within the hybrid zone and increases to high frequency (Figure 2). This could be a reticulation event in the sense that it happens after two races come into secondary contact, and the new allele could be a recombinant of alleles characterising the two races or could arise in relation to mutagenic processes associated with hybridisation. It is also possible that the new allele could arise by processes unrelated to hybridisation and could also occur in hybrid zones formed by primary contact.

Considering the races that may form hybrid zones, including staggered hybrid zones, one characteristic that may define such races is their chromosomal complement. In other words, races may differ through one or multiple chromosomal rearrangements. In hybrid zones between chromosomal races each rearrangement represents a character that is expected to show clinal variation and multiple chromosomal clines may show a coincident or staggered distribution.

There are several reasons why the chromosomal hybrid zones of certain species may be particularly good models to study staggered clines.

First, the availability of such systems. There are many examples of species subdivided into chromosomal races, and often there will be multiple chromosomal differences between those races. There is a tendency within species or among closely related species for the same type of chromosomal rearrangement to affect multiple chromosomes within the karyotype (e.g. multiple pericentric inversions); a process termed ‘karyotypic orthoselection’ [5]. So, there may be multiple clines of the same type of chromosomal rearrangement in multiple hybrid zones of the same species, allowing many comparisons.

Second, the simplicity of such systems. There is an expectation that heterozygotes for chromosomal rearrangements will have reduced fertility due to meiotic aberrations [4], and so the properties of chromosomal hybrid zones, including clinal staggering, can be considered in those terms. It should be emphasised that there is clear evidence that the meiotic aberrations reflect abnormal chromosomal behaviour *per se*, rather than merely some genic changes linked to chromosomal rearrangements (although genic factors may be an influence) [4]. It is expected therefore that chromosomal hybrid zones will be maintained by hybrid unfitness on these chromosomal grounds. There is a strong theoretical underpinning behind the study of zones maintained by hybrid unfitness [1,6].

Third, the importance of such systems. Chromosomal rearrangements have been implicated in speciation and their study in hybrid zones may lead to insights in this respect. Chromosomal rearrangements may be involved in speciation in two ways [7,8,9]. Firstly, because of the reduced fertility associated with chromosomal heterozygotes, this may cause reduced gene flow across the hybrid zone and/or selection leading to assortative mating (‘reinforcement’) [10]. Secondly, recombination may be suppressed in the vicinity of the rearrangement, again leading to reduced gene flow in that part of the genome, allowing the build-up of incompatibilities [7,11].

Not only are there clear examples of staggering of chromosomal clines in hybrid zones, there is also evidence that such staggering does result in the formation of new races. In a recent phylogenetic analysis of chromosomal races in the common shrew *Sorex araneus* [12], the number of evolutionary steps to generate the full set of races was dramatically reduced when zonal raciation by reticulate evolution was allowed. That analysis suggested that 19 out of 72 known chromosomal races in the common shrew may have been generated in this way.

Like the common shrew, the western European subspecies of the house mouse (*Mus musculus domesticus*) is characterised by numerous chromosomal races that make contact and hybridise [13,14]. Also, the types of chromosomal rearrangement involved are the same as in the common shrew. The ancestral karyotype of the house mouse consists of 40 acrocentric chromosomes. The various chromosomal races of the house mouse are characterised by different combinations of metacentric chromosomes formed by two types of chromosomal rearrangement: Robertsonian fusions (where pairs of ancestral acrocentrics fuse at their centromeres) and whole-arm reciprocal translocations (WARTs; the subsequent swapping of chromosome arms between metacentrics or between metacentrics and acrocentrics) [14].

There has not been such a rigorous range-wide phylogenetic study on the chromosomal races of the house mouse as that carried out in the common shrew (though this is in progress [15]), but already there are indications that once again zonal raciation reduces the number of evolutionary steps [12,14].

In this article we will review our current knowledge of staggered hybrid zones involving the chromosomal races of the house mouse. We will also include previously unpublished data where relevant and we will consider possible future work. There is considerable uncertainty in our current knowledge, which we highlight in the hope to stimulate further decisive studies. Clearly, the genomic tools available for the house mouse provide plentiful opportunities for the future. For example, a mouse diversity genotyping array that can simultaneously assay 600,000 single nucleotide polymorphisms (SNPs) has recently become available commercially [16], allowing unprecedented analysis of genomic differences at a population genetic level.

We will focus the forthcoming sections on four specific mouse hybrid zones that we have worked on and use them as case studies to investigate various aspects of non-coincidence of chromosomal clines. In the final section we will extend from this survey to other studies on chromosomal hybrid zones in the house mouse, to generate a synthesis with a focus on reticulate evolution and speciation.

## 2. A staggered hybrid zone arising through primary contact? An area of chromosomal polymorphism near Barcelona, Spain

House mouse populations occupying an area of over 5000 km^2^ in the vicinity of Barcelona are characterised by polymorphism for the following metacentric chromosomes: 3.8, 4.14, 5.15, 6.10, 7.17, 9.11 and 12.13 (where metacentric x.y consists of the ancestral acrocentrics x and y joined at the centromere) [17,18,19]. Thus, each of these chromosome arm combinations may occur in individuals as homozygous acrocentric, heterozygous or homozygous metacentric, with observed diploid numbers ranging from 27 to 40. The highest frequencies of all the metacentrics occur in the same general area about 25 km west of the city of Barcelona [17,18,19]. This has been defined in the past as the location of the ‘Barcelona chromosomal race’ [18], but as is clear from a massive karyotyping effort (451 mice [20]), in fact there is no population among those studied that is ‘fixed’ for all metacentrics or any combination of metacentrics. Indeed, no mouse that is homozygous metacentric for all seven metacentrics (*i.e.*, 2n = 26) has been found, and in particular the frequency of 7.17 never exceeds 0.14 in populations [19]. Despite the absence of a clear Barcelona chromosomal race, chromosomal clines have been defined for five of the metacentrics along a transect from the region of high metacentric frequency towards the periphery where mice are of the standard 40-chromosome acrocentric race [18]. There is clear non-coincidence of the clines (Table 1) and likelihood ratio tests showed that seven out of ten pairwise comparisons were statistically significant [18]. However, the metacentric clines tend to be shallow rather than steep, consistent with the tendency for populations to be polymorphic rather than fixed for particular metacentrics [18].

**Figure 3:**
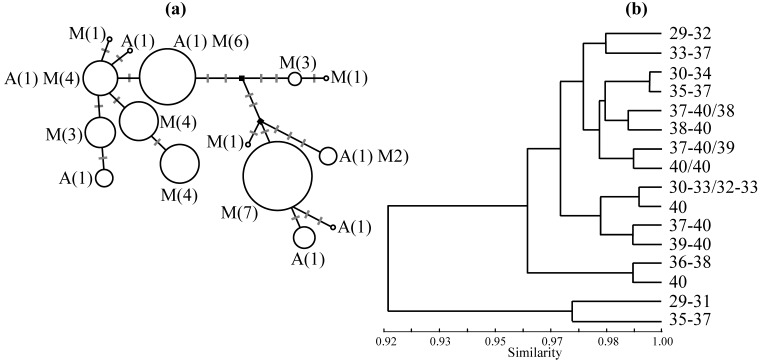
Genetic studies in the house mouse investigating the association of molecular markers and karyotype, based on specimens from 20 localities sampled by us [18] in the area of chromosomal polymorphism near Barcelona. **(a)** A median-joining network [23], as produced in NETWORK (http://www.fluxus-engineering.com), illustrating the relationship amongst the 15 mitochondrial DNA haplotypes including the left end of the D-loop (251 bp) and the flanking Pro-tRNA (54 bp) scored in 83 individuals. For each haplotype, details are given of the number of populations with that haplotype characterised solely by the standard acrocentric karyotype (A) or characterised by at least some metacentric karyotypes (M). Mutation steps are depicted along the branches linking haplotypes. The black squares represent median vectors. The size of each circle is proportional to the frequency of the particular haplotype in the sample. **(b) **An UPGMA phenogram produced in BIOSYS-1 [24] using Nei’s coefficient of genetic identity [25] based on genotypes for six variable allozyme loci (*Amy1*, *Got2*, *Gpi1*, *Idh1*, *Mod1* and *Pgm1*), scored in 126 individuals. For each sample, or combination of two samples (indicated by a slash), used in the analysis, the range of diploid numbers is indicated.

Metacentric mice and the standard acrocentric race in the vicinity of Barcelona are not distinguished by mitochondrial (mt) DNA or allozyme markers (Figure 3). This suggests, together with results from morphological data [21], that this is a primary contact; that a population near Barcelona accumulated metacentrics without a substantial period of geographic isolation and that the staggering of chromosomal clines may reflect some combination of time of formation of the metacentrics and the vagaries of population processes and genetic drift. However, with future genomic data, signatures of selective processes may be identified and one conundrum solved: if there was not a ‘race’ formed in geographic isolation, why are the distributions of all seven metacentrics centred in the same general area? It may be that rapid chromosomal evolution in a very short period of isolation may best explain the pattern observed, though mouse population history should relate to human population history [22] and there is no obvious historical basis for the temporarily isolated population suggested.

**Table 1:** The cline centres (with 95% confidence intervals) for five metacentrics in the Barcelona area showing staggered clines [18].

Metacentric	Cline centre (km)
6.10	12 (8 - 15)
9.11	25 (21 - 28)
5.15	28 (22 - 33)
12.13	31 (26 - 37)
4.14	37 (31 - 45)

## 3. A staggered hybrid zone arising through secondary contact? The John o’Groats-standard hybrid zone in Scotland

**Figure 4:**
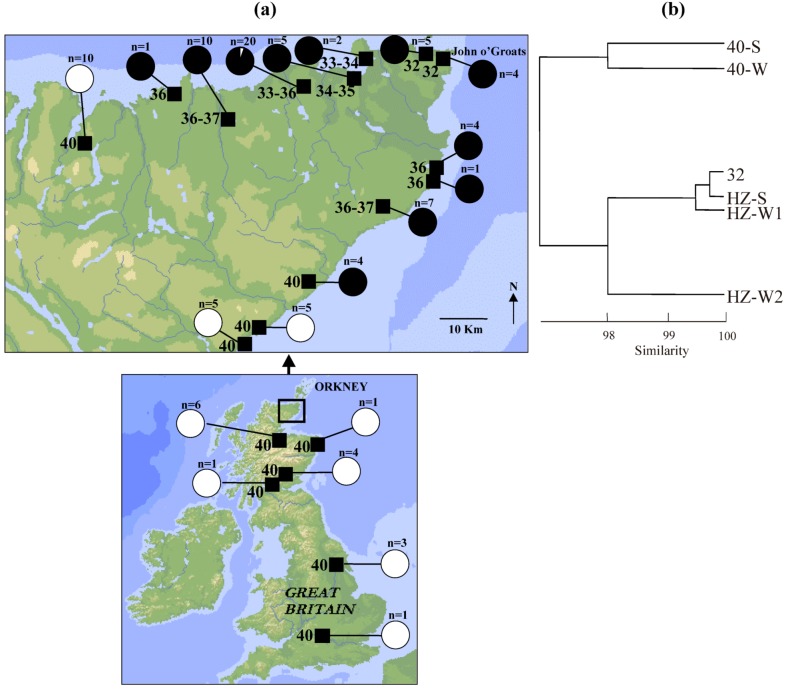
Genetic data relating to the John o’Groats-standard chromosomal hybrid zone in the house mouse. **(a)** Map showing diploid chromosome numbers and mtDNA clades from localities in mainland Britain, and particularly the extreme tip of northern Scotland, with transects to the west and south of John o’Groats across the hybrid zone [22,26,27]. Pies show the proportion of individuals of the Orkney mtDNA clade (in black) based on a sample size indicated. **(b)** A UPGMA phenogram using Nei’s coefficient of genetic identity [25] based on twenty allozyme loci (*Aco1*, *Acp1*, *Ak1*, *Amy2*, *Ck1*, *Es10*, *Got1*, *Got2*, *Gpi1*, *Idh1*, *Idh2*, *Ldh1*, *Ldh2*, *Mod1*, *Mor1*, *Mor2*, *Mpi1*, *Np1*, *Pgm1* and *Pgm2*; previously unpublished data), showing populations from the John o’Groats-standard hybrid zone, distinguishing between the pure race (2n = 32 or 40) and intermediate (HZ) populations and whether they come from the southern (S) or western (W) transect. Sample sizes for each population are as follows: ‘40-S’: 29; ‘40-W’: 9; ‘32’: 18; ‘HZ-S’: 7; ‘HZ-W1’: 42; ‘HZ-W2’: 11.

Within the British Isles there is a mitochondrial (mt) DNA clade with an extreme northerly and westerly distribution which matches well to the sphere of influence of the Norwegian Vikings, and it is likely that the mice of this ‘Orkney’ clade attained their distribution through accidental transport by the Vikings [22]. Mice of this clade in the northern tip of mainland Scotland and some of the Orkney islands immediately to the north of that are characterised by karyotypes with metacentric chromosomes, while mice from neighbouring parts of mainland Scotland have mtDNA haplotypes belonging to different clades and are characterised by the standard all acrocentric karyotype (Figure 4a) [17,22,26,27,28]. Therefore, it appears that the metacentric chromosomes arose in Orkney clade mice and that the chromosomal hybrid zone in northern mainland Scotland represents a secondary contact between mice of that clade and mice of other origin (with some introgression on contact: Figure 4a). Further support of this comes from allozyme data (Figure 4b), which again indicates the distinctiveness of the mice of the standard acrocentric karyotype in this region.

**Figure 5:**
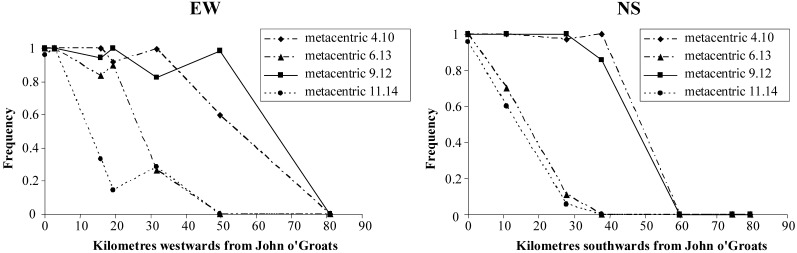
Transects across the John o’Groats-standard hybrid zone to the west and south of John o’Groats, showing metacentric clines [26,27,29].

Mice at John o’Groats have a 32-chromosome karyotype (Figure 4a) including the following metacentric chromosomes in a homozygous state: 4.10, 9.12, 6.13, 11.14. This is the John o’Groats metacentric race and it is the contact of this race and the standard acrocentric race that forms a staggered structure (Figure 5). Both to the west and particularly the south of John o’Groats, there is an area with a high frequency of 2n = 36 mice, homozygous for metacentrics 4.10 and 9.12. There is further staggering evident in the westerly but not southerly direction.

There are various imponderables about this zone that would benefit greatly from genomic analysis. Given the secondary contact, a reasonable scenario would be that the 32-chromosome race met the 40-chromosome race, the hybrid zone formed and staggering occurred after that. However, to demonstrate this, it would be desirable to have a genetic signal showing introgression of acrocentric chromosomes from the standard race into the metacentric race. This is currently not available.

Instead, there is a widespread 36-chromosome form that makes contact with the standard race. The two metacentric chromosomes that characterise that form, 4.10 and 9.12, are also found on the Orkney islands [17] and therefore could have been the first metacentrics to form within the John o’Groats race, with chromosomes 11.14 and 6.13 (not found on the Orkney islands [14]) arising later on. So, there is a possibility that the non-coincidence of chromosomal clines in the John o’Groats-standard hybrid zone reflects a primary contact, and that the secondary contact is merely between the 36-chromosome form and the standard race.

## 4. Zonal raciation in the Upper Valtellina hybrid zone in Italy?

Within the 20 km section of alpine valley in northern Italy known as ‘Upper Valtellina’ there are two chromosomal races that have not been observed elsewhere: IMVA and IUVA [14,30,31]. Flanking these two races and extending into neighbouring areas are two other races: CHPO and ILVA [31,32]. On grounds of karyotype, phylogeny and distribution [14,31], the IMVA and IUVA races appear to be the products of hybridisation of the CHPO and ILVA races and therefore examples of ‘zonal raciation’. In Table 2 it can be seen that both the IMVA and IUVA races have a combination of chromosomes that could be derived from the CHPO and ILVA races. Thus, an F_1_ hybrid between the CHPO and ILVA races would form a chain-of-five configuration (2-2.8-8.12-12.10-10) and a chain-of-three configuration (7-7.18-18). Crosses between these hybrids could generate all the karyotypes shown in Table 2.

**Table 2:** Race-specific metacentrics and acrocentrics of chromosomal races in Upper Valtellina.

Race	Race-specific metacentrics and acrocentrics							
CHPO	2		8.12		10	7		18
ILVA		2.8		10.12			7.18	
IMVA	2		8.12		10		7.18	
IUVA		2.8		10.12		7		18

Identifying the IMVA and IUVA forms either as populations within a staggered hybrid zone or as new independent races is somewhat arbitrary. Pialek *et al*. [14] recognise this and give the same status to the 36-chromosome form within the John o’Groats hybrid zone as to the IMVA and IUVA forms. However, the IMVA and IUVA forms occur in a patchy fashion around the contact of the CHPO and ILVA races; this is not a neat, linear hybrid zone [32].

It is difficult to visualise the formation of the IMVA and IUVA races other than in terms of secondary contact and reticulate evolution, but molecular studies using chromosome markers mapped to race-specific chromosomes are being conducted to confirm this [33]. Selective processes may be involved in the formation of these races in the patchy setting of the hybrid zone. On hybridisation of the CHPO and ILVA races within a population patch, there is an expectation that *either* IMVA *or* IUVA homozygotes would be favoured because they cannot produce offspring that are so highly heterozygous, and therefore so unfit, as the CHPO x ILVA F_1_ hybrids [10].

## 5. ‘Staggered clines within one locus.’ The Madeira hybrid zone

**Figure 6:**
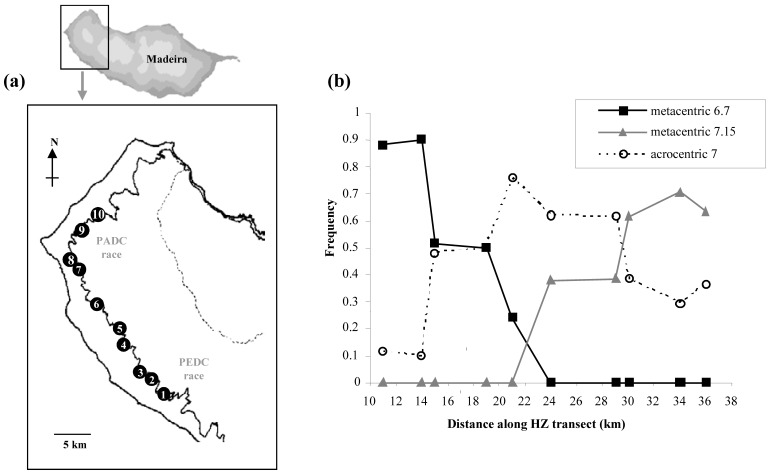
The Madeira house mouse hybrid zone between the PEDC and PADC chromosomal races, based on data in Nunes *et al*. [34]. **(a)** Map showing the ten collection sites that form a transect between the two races. **(b)** Frequencies of the three ‘alleles’ of chromosome 7 along the transect.

The PEDC chromosomal race of house mouse on the Atlantic island of Madeira is characterised by the metacentric 6.7 and makes contact with the PADC race characterised by metacentric 7.15 (Figure 6) [34]. Because of the involvement of chromosome 7 in two different metacentrics, these can be viewed as equivalent to two different alleles of one locus. The clines for 6.7 and 7.15 are staggered because of the presence of a third ‘allele’: chromosome 7 in an acrocentric state. This allele occurs at a high frequency leading to domination at the centre of the zone by individuals homozygous for chromosomes 6, 7 and 15. As in the situation with Upper Valtellina, the presence of acrocentrics at the centre of the hybrid zone may be a response to selection. The F_1_ hybrid between the PEDC and PADC races will form a chain-of-four configuration at meiosis (6-6.7-7.15-15). There is an expectation that individuals homozygous for chromosomes 6, 7 and 15 will be favoured because they cannot produce such heterozygous and unfit offspring as the PEDC x PADC F_1_ hybrids [4].

The origin of acrocentric 7 is intriguing. From the chromosomal phylogenies that have been constructed, it can be inferred that metacentric 6.7 was formed by Robertsonian fusion well before the evolution of race PEDC, and that during formation of race PADC metacentric 7.15 evolved from 6.7 by a WART [12,35]. It is not to be expected that the ancestral state would still be retained by either race. Nor is it expected that chromosome 7 would arise by Robertsonian fission, because this is believed unlikely to occur in house mice [36]. There are standard acrocentric mice on Madeira [37,38] and so it is possible that movement of these mice around the island introduces acrocentric chromosomes. It is known that standard acrocentric mice interbreed successfully with one of the metacentric races on Madeira [37,38]. Ongoing studies with chromosome specific molecular markers will help identify the origin of acrocentric 7 in the PEDC-PADC hybrid zone [39].

## 6. Summary and Synthesis

The above survey of staggered hybrid zones in the house mouse indicates a variety of different hybrid zone structures and a various modes of origin. Hybrid zones examined by other researchers reinforce what we have described:

In Belgium the BBEL race characterised by 4.12 and 5.10, in Germany the DSTJ race with 5.15 and 13.14 and in Denmark the DKEA race with 2.5, 3.8 and 6.9 show staggered clines similar to those seen in the John o’Groats-standard hybrid zone, with intermediate homozygous forms at high frequency in parts of the hybrid zone [4,14,40,41,42,43]. As for the John o’Groats-standard hybrid zone, the degree of staggering of the different chromosomes differs according to the transect direction across the Belgian and German hybrid zones [40,41,42]. In northern Italy, the most widely prevalent and presumably the first formed metacentric among chromosomal races there, 16.17, has a wider distribution than other metacentrics at the contact between the IBIN metacentric race and the standard acrocentric race [14,44]. Again this has similarities to the situation described for the John o’Groats-standard hybrid zone. In France there is a second case of ‘staggered clines within one locus’: the FBIS race with metacentrics 5.7 and 10.14 and the FSAN race with metacentric 5.10, and the acrocentrics 5 and 10 at high frequency in the intermediate area [14,45]. Therefore, for chromosome 5 there are three alleles, metacentrics 5.7 and 5.10 and acrocentric 5, and for chromosome 10 the three alleles are metacentrics 5.10 and 10.14 and acrocentric 10. From an allozyme analysis there is no clear evidence that the acrocentrics 5 and 10 were introduced into the hybrid zone, but the authors acknowledge that further molecular work is necessary to confirm this.

The data that are available for staggered hybrid zones in the house mouse are largely chromosomal. The molecular analyses that have been conducted so far have been small scale and ‘old fashioned’, and therefore should be seen as preliminary, although substantive studies with mapped loci are in progress for both the Valtellina and Madeira hybrid zones [33,39]. Genomic analyses have a huge potential to identify the origin of homozygous forms within staggered hybrid zones and to illuminate other aspects of these systems. This review article should be seen as a baseline on which those genomic studies can build.

Despite the need for more data on staggered hybrid zones in the house mouse, there are two generalities that can be made:

a) It appears that staggering may sometimes reflect time of formation of metacentrics, with earlier formed metacentrics more widespread than other metacentrics. This form of staggering relates to primary contact. Whether metacentrics are spreading through the ancestral population through some sort of selective advantage or meiotic drive as posited in the stasipatric model of White [46], or whether population processes and genetic drift can explain the pattern, is unclear. In another ‘classic’ case where White applied his model, the Australian grasshopper *Vandiemenella*, a stasipatric process has been discredited [47].
b) Non-coincident clines tend to occur in hybrid zones where there are only a few chromosomal differences between the hybridising races. Other hybrid zones that have been described in northern and central Italy and Tunisia where there is a contact between a 22 or 24 chromosome race and the standard 40 chromosome race or where there are massive differences between metacentric races, there tends not to be staggering associated with the hybrid zone [33,48,49,50,51], as already noted by Searle [4]. The following chromosomal explanation may be suggested: Where there are many chromosomal differences, F_1_ hybrids are very unfit and selective forces tend to hold the clines together [4,52]. Thus, hybrid zones with fewer chromosomal differences are less constrained by such forces, and so staggering is ‘permitted’ (though this does not *necessarily* occur: see Mitsainas *et al*. [53]).

The contribution of staggered hybrid zones in the house mouse to reticulate evolution is surprisingly uncertain. This is exemplified by the John o’Groats-standard hybrid zone, where there appears to have been secondary contact between the standard acrocentric race and metacentric populations, but it has not been confirmed that the staggering of chromosomal clines relates to that secondary contact.

Considering other species than the house mouse: it should be emphasised that not only are there also examples of homozygous forms that clearly have a hybrid origin, but those homozygous forms may additionally show adaptive differences from the parental races. This may be important in speciation, e.g. in sunflowers [54]. Such adaptive features are not known for any of the homozygous forms found in hybrid zones in the house mouse.

Does this mean that staggered hybrid zones in the house mouse are irrelevant to reticulate evolution and speciation?

Actually, there is one case in the house mouse for which there is good evidence for reticulate evolution: the generation of the IMVA and IUVA races in Upper Valtellina. Detailed phylogenetic analysis of house mouse chromosomal races should reveal other examples. For the similar chromosomal system in the common shrew, phylogenetic analysis suggests very substantial reticulate evolution [12].

But more important than adding up the number of examples of reticulate evolution, is to rethink the relevance of this process. In the house mouse, the metacentric races are highly localised forms; like ‘islands’ within a ‘sea’ created by the standard acrocentric race [13,14]. Often there may be a primary contact between a metacentric race and the standard acrocentric race, and staggering of metacentric clines in the hybrid zone may relate to time of formation of the metacentric chromosomes. Under these circumstances the homozygous forms within staggered hybrid zones are earlier stages in the evolution of the metacentric race involved in the hybrid zone. However, if a homozygous form from a hybrid zone like that becomes a new race, isn’t it in some ways a semantic point as to whether it is the product of reticulate evolution or not? The race will have a combination of the characteristics of the hybridising races in the hybrid zone from which it derives, just like a race that arises by reticulate evolution. There is no qualitative difference thereafter in the expected evolutionary trajectory of races produced by zonal raciation from a primary or secondary contact.

There are other grounds for not focussing too closely on primary *versus* secondary contact and whether races generated by zonal raciation are examples of reticulate evolution or not: the John o’Groats-standard hybrid zone illustrates how hybrid zones may attain their characteristics through a mixture of primary and secondary contact, complicating interpretation.

The important point is that zonal raciation contributes to the diversity of chromosomal races that are present in a species like the house mouse. Whatever way new chromosomal races are produced in hybrid zones, they add to the stock of chromosome races produced ‘*de novo*’ and increase the diversity of chromosomal races present in the organism. This is the important issue with regards the possibility of speciation since each one of those races has the potential to become a new species.

Speciation is an infrequent phenomenon that may often involve a quirky set of circumstances. If ‘races’ can be viewed as incipient species, then the more races there are within a species, the greater the likelihood that there will be a speciation event.

Races defined by karyotype may be particularly important as precursors of new species. As explained earlier, the chromosomal difference shown by a race relative to other races can contribute to divergence and reproductive isolation in two ways: through reduced fertility of the chromosomally heterozygous hybrids or through recombination suppression in the vicinity of the rearrangement in those hybrids. These processes may lead to interrupted gene flow between the races or reinforcement. The extent of reproductive isolation may be enhanced through further accumulation of chromosomal differences, possibly promoted by the prior presence of chromosomal rearrangements [13]. It may be that geographic isolation of chromosomal forms is particularly likely to lead to new species, because the genic divergence in isolation may combine in a synergistic way with the chromosomal differences in promoting infertility [55].

There is evidence that chromosomal rearrangements may help to retain or promote divergence during speciation. In the common shrew there are two forms, the Vaud race of the common shrew ‘*sensu stricto*’ *Sorex araneus* and the Valais shrew *Sorex antinorii*, which come into contact and very occasionally hybridise. Basset *et al*. [56] and Yannic *et al*. [57] showed that there is greater divergence between the Vaud *S. araneus* and *S. antinorii* for loci on chromosomes that differ between the two species than for loci on chromosomes that are common to the two species. It is probable that the genic divergence occurred in allopatry, with better protection of that divergence by chromosomes involved in chromosomal rearrangements than by chromosomes that are not.

In the house mouse, there is considerable evidence of reduced fertility and even complete sterility in hybrids between chromosomal races, relating to their chromosomal differences, as reviewed in Searle [4] and Piálek *et al*. [10]. Among mouse chromosomal races there have been two examples of neighbouring races where, despite substantial collections, no hybrids have been found, suggesting behavioural barriers to interbreeding [30,49].

Taking a broader perspective, it is notable that closely related species very often differ by chromosomal rearrangements [58]. Of course these chromosomal differences could evolve after speciation, but actually it is easier to envisage species-specific karyotypes as being present at the time of origin of species rather than spreading through the species, or emerging in some other way (e.g. fixation during later population bottlenecking). That suggests that the karyotypic differences between species may often be already present as distinctive chromosomal races within the ancestral species.

Hence we argue that chromosomal races may actually be more important in the speciation process than is widely realised and that zonal raciation may contribute to speciation by increasing the ‘stock’ of chromosomal races. This all relates to staggered hybrid zones, because it is through staggering of chromosomal clines that zonal raciation may occur.

Given this potential importance of staggered chromosomal hybrid zones, we wish to encourage further research on them, and the house mouse is clearly a particularly good model. There are excellent opportunities for studying why staggered chromosomal hybrid zones should come about, determining the pattern of gene flow within them and establishing their outcomes in terms of generation of new races. A theoretical, simulation modelling approach (such as that adopted by Hatfield *et al*. [59]) is an important tool for the first question, and empirical genomic approaches using, for instance, the mouse SNP array [16], are clearly of value in the other two topics. Being able to document in extraordinary detail the gene exchange between taxa is one of the most substantial achievements of the genomic revolution, as illustrated by examples such as humans-Neandertals [60], and there has been a long tradition to use molecular markers to identify new forms in plants that have arisen following hybridisation [61,62] which could be followed in the house mouse. The grounding we provide here on staggered chromosomal hybrid zones can be taken much further with such approaches.
